# PD-L1 expression and its correlation with clinicopathological and molecular characteristics in Chinese patients with non-small cell lung cancer

**DOI:** 10.1097/MD.0000000000036770

**Published:** 2024-02-23

**Authors:** Jindong Guo, Haibin Yuan, Yimin Zhu, Zhiyuan Che, Bei Zhang, Ding Zhang, Ying Zhou, Liwen Xiong

**Affiliations:** a Department of Radiation Oncology, Shanghai Chest Hospital, Shanghai Jiao Tong University, Shanghai, China; b Department of Emergency, Shanghai Chest Hospital, Shanghai Jiao Tong University, Shanghai, China; c Department of Pulmonary and Critical Care Medicine, Shanghai Sixth People’s Hospital Affiliated to Shanghai Jiao Tong University School of Medicine, Shanghai, China; d Department of Health Policy and Management, Bloomberg School of Public Health, Johns Hopkins University, Baltimore, USA; e The Medical Department, 3D Medicines Inc., Shanghai, China; f Department of Oncology, Shanghai East Hospital, Tongji University, Shanghai, China; g Department of Pulmonary Medicine, Shanghai Chest Hospital, Shanghai Jiao Tong University, Shanghai, China.

**Keywords:** EGFR, immunotherapy, KRAS, NSCLC, PD-L1

## Abstract

Little is known about the relationship between programmed cell death-ligand 1 (PD-L1) expression and histologic and genetic features in real-world Chinese non-small cell lung cancer patients. From November 2017 to June 2019, tumor tissues were collected from 2674 non-small cell lung cancer patients. PD-L1 expression was detected with immunohistochemistry using the 22C3 and SP263 antibodies, and patients were stratified into subgroups based on a tumor proportion score of 1%, 1% to 49%, and ≥ 50%. Genetic alterations were profiled using targeted next-generation sequencing. In the total population, 50.5% had negative PD-L1 expression (tumor proportion score < 1%), 32.0% had low-positive expression (1%–49%), and 17.5% had high-positive expression (≥50%). The PD-L1 positive rate was 39.0% in squamous cell carcinomas and 53.6% in adenocarcinomas. PD-L1 expression was higher in squamous cell carcinomas (*P* < .001) and lower in adenocarcinomas (*P* < .001). Of the overall patient population, 11.2% had Kirsten rat sarcoma viral oncogene (KRAS) mutations, 44.9% had epidermal growth factor receptor (*EGFR*) mutations, 2.1% had *BRAF V600E* mutations, 0.3% had *MET* exon 14 skipping mutations, 5.4% had *anaplastic lymphoma kinase* translocations, and 0.9% had *ROS proto-oncogene 1* translocations. Patients carrying *ROS proto-oncogene 1* translocations (*P* = .006), *KRAS (P* < .001), and *MET (P* = .023) mutations had significantly elevated expression of PD-L1, while those harboring *EGFR (P* < .001) mutations had lower PD-L1 expression. In our study, PD-L1 expression was significantly higher in squamous cell carcinomas and lower in adenocarcinomas, and was positively associated with *MET* and *KRAS* mutations, as well as the wild-type *EGFR* gene state. Nonetheless, additional studies are needed to further validate those associations and determine the clinical significance for immune checkpoint inhibitors of these factors.

## 1. Introduction

Lung cancer is a leading cause of cancer-related deaths in China,^[1,2]^ with non-small cell lung cancer (NSCLC) accounting for approximately 85% of cases.^[3]^ A recent implementation of immune checkpoint inhibitors (ICIs), such as antibodies against programmed cell death-1 (PD-1) and programmed cell death-ligand 1 (PD-L1), has shifted the therapeutic paradigm for NSCLC. Multiple anti-PD-1/PD-L1 therapies have been approved for first- and second-line treatment patients with advanced NSCLC.^[4–7]^ In the first-line setting, pembrolizumab monotherapy demonstrated superior efficacy compared to chemotherapy in patients with advanced or metastatic NSCLC who tested negative for epidermal growth factor receptor (*EGFR*) sensitizing mutations or anaplastic lymphoma kinase (*ALK*) fusions and exhibited a PD-L1 tumor proportion score (TPS) of ≥ 1%.^[4,5]^

In light of the fact that PD-L1 expression is the only US Food and Drug Administration-approved predictive biomarker for anti-PD-1/PD-L1 treatment of NSCLC, it is prevalent to investigate factors that affect PD-L1 expression and analyze their application prospects for ICIs. A meta-analysis showed that PD-L1 expression in NSCLC patients positively correlated with smoking, male gender, an adenocarcinoma histology, and wild-type *EGFR*, *ALK*, ROS proto-oncogene 1 (*ROS1*), and Kirsten rat sarcoma viral oncogene *(KRAS*) genes.^[8]^ However, another meta-analysis conducted by Huang et al demonstrated that survival benefits from ICIs in NSCLC were not significantly associated with histology, central nervous system metastases, age, gender, or performance status.^[9]^ Several additional studies have also demonstrated that the regulation of PD-L1 expression in NSCLC was influenced by oncogenic drivers, including but not limited to *EGFR, ALK, ROS1, KRAS, TP53, MET*, and *PIK3CA*.^[10,11]^ Apart from single gene alteration, some investigations have endeavored to identify potential correlations between tumor mutational burden (TMB), a quantitative indicator of the prevalence of genic mutations, and PD-L1 levels. However, no conclusive evidence has been obtained thus far.^[12,13]^

Although several western studies provided evidence regarding the relationship between molecular phenotypes, clinicopathological factors, and PD-L1 expression in NSCLC patients, it is widely acknowledged that the genomic profiling of Chinese NSCLC is significantly different from that of TCGA, especially the mutation rates of *EGFR*, which can be up to approximately 50%.^[14,15]^ To date, there are few relevant studies and evidence regarding the relationship between the above factors and PD-L1 expression in Chinese NSCLC patients. In this work, we performed next-generation sequencing and PD-L1 immunohistochemistry (IHC) on tumor tissues obtained from 2674 Chinese NSCLC patients. The objective was to explore the correlation between clinicopathologic, genomic patterns and PD-L1 expression, as well as their prognostic value in Chinese patients.

## 2. Materials and methods

### 2.1. Patient and samples

Patients diagnosed with NSCLC at our institution from November 2017 to June 2019 were retrospectively included (Fig. S1, Supplemental Content, http://links.lww.com/MD/L190, which demonstrates the patient selection flowchart). The inclusion criteria were patients between the ages of 18 and 90, male or female; patients histologically confirmed NSCLC; patients had PD-L1 and 381-genes NGS testing results. The exclusion criteria were as follows: Patients only with plasma sample tested and patients with formalin-fixed paraffin-embedded (FFPE) tumor tissue block that older than 5 years. After applying both inclusions and exclusion criteria, 2674 patients with NSCLC were included in the study. The study was approved by the Ethics Committee of the Institutional Review Boards at Shanghai Chest Hospital.

### 2.2. PD-L1 IHC

FFPE tumor samples were subjected to PD-L1 IHC using the PD-L1 IHC 22C3 pharmDx assay (Agilent Technologies; catalog number, SK006) or the PD-L1 IHC SP263 assay (Roche Diagnostics GmbH; catalog number,740-4907/07208162001). The staining for 22C3 was performed on a Dako Link-48 autostainer system at Teddy Clinical Research Laboratory (Shanghai) Limited, and the staining for SP263 was performed on a Roche BenchMark Ultra platform at QIAGEN (Suzhou) Clinical Lab, following the manufacturer instructions. The proportion of tumor cells showing membranous staining was scored as TPS by the technical experts and then classified as negative, low-positive, or high-positive PD-L1 expression based on the TPS score of < 1%, 1% to 49%, or ≥ 50%.

### 2.3. Genetic profiles and TMB estimation

Next generation sequencing targeting 381 cancer-related genes was conducted at 3D Medicines, Inc.,. Genomic DNA was extracted from FFPE tumor and paired normal tissue samples. TMB was defined as the number of synonymous and non-synonymous somatic mutations, stop gain/loss, and splicing variants and indels per Mb in examined coding regions.

### 2.4. Statistical analyses

The prevalence of PD-L1 positivity was compared between subgroups using Chi-square analysis or Fisher exact test, as appropriate. All analyses were performed using R version 3.6.1, and *P* values <.05 were considered statistically significant.

## 3. Results

### 3.1. PD-L1 expression and clinical characteristics

Of the 2674 NSCLC patients enrolled, 1322 (49.5%) were positive for PD-L1 expression (TPS ≥ 1%), with 467 (17.5%) showing strong positive (TPS ≥ 50%) (Table 1). There were no significant differences in age between patients with negative, low, and high-positive PD-L1 expression (*P* = .315). However, the prevalence of PD-L1 expression was found to be higher in women (438/1000, 58.4%) than in men (884/1674, 41.6%), with a significant difference (*P* < .001). With regards to sampling methods and sites, it was found that resected samples showed significantly higher levels of PD-L1 positivity than biopsied samples (57.7% vs 42.3%, *P* < .001). Additionally, PD-L1 expression was mainly distributed in samples collected from metastatic sites (79.7%) as opposed to those from primary sites (20.3%, *P* = .002). Furthermore, freshly prepared samples were found to have a significantly higher proportion of PD-L1 expression (77.1%) than archived samples (77.1%, *P* < .001).

**Table 1:** Correlations between clinicopathological features and PD-L1 tumor proportion score.

Characteristic		PD-L1 ≥ 50% n = 467 (17.5%)	PD-L1 1%–49% n = 855 (32.0%)	PD-L1 < 1% n = 1352 (50.5%)	No. of cases n = 2674	*P* value
Age (yr)	<35	8 (1.7%)	20 (2.3%)	33 (2.4%)	61 (2.3%)	.3154
	35–50	52 (11.1%)	121 (14.2%)	198 (14.6%)	371 (13.9%)	
	>50	407 (87.2%)	714 (83.5%)	1121 (82.9%)	2242 (83.8%)	
Sex	Female	138 (29.6%)	300 (35.1%)	562 (41.6%)	1000 (37.4%)	<.0001
	Male	329 (70.4%)	555 (64.9%)	790 (58.4%)	1674 (62.6%)	
Sampling type	Biopsy	338 (72.4%)	541 (63.3%)	780 (57.7%)	1659 (62.0%)	<.0001
	Resection	129 (27.6%)	314 (36.7%)	572 (42.3%)	1015 (38.0%)	
Sampling site	Primary	335 (71.7%)	667 (78.0%)	1078 (79.7%)	2080 (77.8%)	.0016
	Metastatic	132 (28.3%)	188 (22.0%)	274 (20.3%)	594 (22.2%)	
Material	Freshly prepared specimens	153 (32.8%)	240 (28.1%)	310 (22.9%)	703 (26.3%)	<.0001
	Archival specimens	314 (67.2%)	615 (71.9%)	1042 (77.1%)	1971 (73.7%)	
Histologic type	Adenocarcinoma	344 (73.7%)	639 (74.7%)	1135 (83.9%)	2118 (79.2%)	<.0001
	Squamous Cell Carcinoma	123 (26.3%)	216 (25.3%)	217 (16.1%)	556 (20.8%)	

The prevalence of PD-L1-positive tumors was 83.9% for squamous cell carcinoma and 16.1% for adenocarcinoma (Table 1). In the squamous cell carcinoma subgroup, 123 patients (26.3%) had strong PD-L1 expression, and 216 (25.3%) had low expression. Patients with squamous cell carcinomas were more likely than those with non-squamous cell histology subtypes to express PD-L1 (*P* < .001). Among patients with adenocarcinoma, 344 patients (26.3%) exhibited high-positive PD-L1 expression. There were no significant differences in the proportions of PD-L1 expression in the adenosquamous and NSCLC—not otherwise specified subgroups.

### 3.2. PD-L1 expression and genomic mutations

#### 3.2.1. Diver mutations.

In addition to PD-L1 IHC staining, all NSCLC tissue samples were sequenced using validated commercial multi-gene next-generation sequencing panels to explore the correlation between oncogenic mutations and PD-L1 expression. Out of the 2674 patients, 1201 (44.9%) carried *EGFR* mutations, 299 (11.2%) carried *KRAS* mutations, 8 (0.3%) carried MET exon 14 skipping mutations, and 56 (2.1%) carried *ROS1* translocations, as shown in Table 2. The study found that tumor PD-L1 expression was more common among patients with wild-type *EGFR* than those mutated for *EGFR* (51.3% vs 48.7%, *P* < .001). Conversely, patients carrying *KRAS* and *MET* mutations had a higher prevalence of PD-L1 positivity than those with wild-type *KRAS* (91.4% vs 8.6%, *P* < .001) and *MET* (99.9% vs 0.1%, *P* < .001), respectively. There was no difference in PD-L1 expression between patients carrying *BRAF V600E* mutation and those with wild-type *BRAF*.

**Table 2:** Correlations between oncogenic driver mutation status and PD-L1 tumor proportion score.

			PD-L1 ≥ 50% n = 467 (17.5%)	PD-L1 1%–49% n = 855 (32.0%)	PD-L1 < 1% n = 1352 (50.5%)	No. of cases n = 2674	*P* value
EGFR mutation		Mutated	135 (28.9%)	371 (43.4%)	694 (51.3%)	1201 (44.9%)	<.001
		Wild-type	332 (71.1%)	484 (56.6%)	658 (48.7%)	1473 (55.1%)	
KRAS mutation		Mutated	93 (19.9%)	90 (10.5%)	116 (8.6%)	299 (11.2%)	<.001
		Wild-type	374 (80.1%)	765 (89.5%)	1236 (91.4%)	2375 (88.8%)	
ALK translocation		Positive	37 (7.9%)	60 (7.0%)	47 (3.5%)	144 (5.4%)	<.001
		Negative	430 (92.1%)	795 (93.0%)	1305 (96.5%)	2530 (94.6%)	
ROS1 translocation		Positive	7 (1.5%)	12 (1.4%)	4 (0.3%)	23 (0.9%)	.006
		Negative	460 (98.5%)	843 (98.6%)	1348 (99.7%)	2651 (99.1%)	
MET Exon 14 Skipping		Positive	4 (0.9%)	3 (0.4%)	1 (0.1%)	8 (0.3%)	.027
		Negative	463 (99.1%)	852 (99.6%)	1351 (99.9%)	2666 (99.7%)	
BRAF V600E mutation		Mutated	4 (0.9%)	8 (0.9%)	8 (0.6%)	20 (0.7%)	.630
	Wild-type		463 (99.1%)	847 (99.1%)	1334 (99.4%)	2654 (99.3%)	

ALK = anaplastic lymphoma kinase, EGFR = epidermal growth factor receptor, KRAS = Kirsten rat sarcoma viral oncogene, ROS1 = ROS proto-oncogene 1.

### 3.3. EGFR and KRAS mutation subtypes

Since aberrations in *EGFR* exons 19 to 21 and *KRAS* G12X were most clinically relevant in NSCLC, the study subsequently focused on mutations occurring in these regions. Of 1201 NSCLC patients with *EGFR* mutations, exon 19 deletions, exon 21 L858R, and exon 20 T790M were identified in 543, 511, and 64 patients, respectively. PD-L1 expression was observed in 63.7% and 66.4% of patients carrying exon 19 deletion and L858R, respectively. Whereas the proportion of PD-L1 expression in patients carrying T790M was 95.2%, less than other *EGFR* mutation subtypes. Additionally, 8 forms of *KRAS* G12X variants were identified in this study: G12A, G12C, G12D, G12F, G12R, G12S, G12V and G12D. The correlation between PD-L1 expression and *EGFR, KRAS* hotspot mutations was summarized in Table 3.

**Table 3:** Correlations between EGFR and KRAS mutation subtypes and PD-L1 tumor proportion score.

		PD-L1 ≥ 50%	PD-L1 1%–49%	PD-L1 < 1%	*P* value
EGFR mutation	DelExon19	50 (26.7%)	168 (32.9%)	325 (36.3%)	<.001
	L858R	62 (33.2%)	148 (29.0%)	301 (33.6%)	
	T790M	4 (2.1%)	17 (3.3%)	43 (4.8%)	
	Other	71 (38.0%)	178 (34.8%)	227 (25.3%)	
KRAS mutation	G12A	8 (10.4%)	10 (14.1%)	9 (9.8%)	.436
	G12C	36 (46.8%)	30 (42.3%)	29 (31.5%)	
	G12D	15 (19.5%)	15 (21.1%)	22 (23.9%)	
	G12F	3 (3.9%)	2 (2.8%)	1 (1.1%)	
	G12R	0 (0%)	0 (0%)	1 (1.1%)	
	G12S	1 (1.3%)	3 (4.2%)	4 (4.3%)	
	G12V	13 (16.9%)	11 (15.5%)	26 (28.3%)	

*Some patients had more than one above mutation and are represented in more than one column.

EGFR = epidermal growth factor receptor, KRAS = Kirsten rat sarcoma viral oncogene, PD-L1 = programmed cell death-ligand 1.

### 3.4. Other mutation genotypes

**Figure 1.**
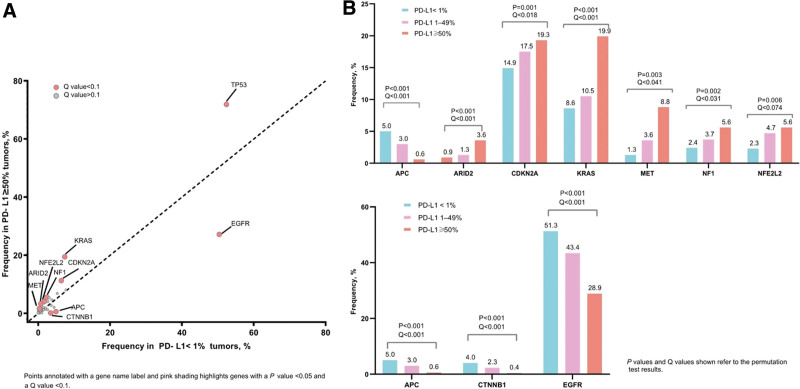
Gene mutations associated with PD-L1 expression levels.

As our panel covered 381 oncogenes, we also analyzed other genetic alterations and their associations with PD-L1 expression. It was found that the PD-L1 negative group, compared with the PD-L1 positive group, exhibited enriched mutations in *APC* (4.7% vs 0.6%, *P* < .001), *CTNNB1* (3.8% vs 0.2%, *P* < .001), *EGFR* (50.4% vs 27.2%, *P* < .001) (Fig. 1). By contrast, the PD-L1 high-positive group, compared to the negative group, was associated with mutations in *TP53* (71.9% vs 52.4%, *P* < .001), *MET* (1.7% vs 0.3%, *P* = .003), *ARID2* (3.4% vs 0.7%, *P* < .001), *NF1* (5.6% vs 2.4%, *P* = .002), *CDKN2A* (11.3% vs 6.4%, *P* = .001), *KRAS* (19.5% vs 7.4%, *P* < .001) and *NFE2L2* (4.1% vs 1.7%, *P* = .006). After correcting for multiple comparisons, all of these enrichments retained significance.

The study further investigated the potential relationship between copy number variance and PD-L1 expression. Interestingly, patients harboring amplification of *JAK2*, *PDC1LG2* (PD-L2), and *CD247* (PD-L1) exhibited high PD-L1 expression levels and enriched in lung squamous carcinoma (Fig. S2, Supplemental Content, http://links.lww.com/MD/L191, which demonstrates the copy number variance associated with PD-L1 expression).

The correlation between mutations in distinct signaling pathways and the level of PD-L1 expression was also investigated. The expression difference of PD-L1 was the most significant in the case of Wnt pathway mutations (3% with PD-L1 positive vs 10% with PD-L1 negative), suggesting that Wnt pathway inhibitors might enhance the efficacy of ICIs by promoting T cell infiltration into tumors (Fig. S3, Supplemental Content, http://links.lww.com/MD/L192, which demonstrates the relationship between the mutations in different signaling pathways and the level of PD-L1 expression).

The mutation burden in lung and other cancers is also considered predictive of benefit for checkpoint inhibition. In this study, the median TMB of the high-positive, low-positive, and negative PD-L1 expression subgroups was 8.1/MB, 6.7/MB, and 5.6/MB, respectively (Fig. S4, Supplemental Content, http://links.lww.com/MD/L193, which demonstrates TMB across PD-L1 expression in tumor).

## 4. Discussion

This work represents a large-scale investigation into the relationship between PD-L1 expression and clinicopathologic and molecular characteristics in Chinese NSCLC patients. Using TPS ≥ 1% as the cutoff, our cohort had a PD-L1-positive rate of 56.1%, which is consistent with previous reports on Asian populations.^[16]^ Currently, pembrolizumab is the first-line treatment for metastatic NSCLC patients with PD-L1 TPS ≥ 50% in some countries, including China. In the present study, 17% of patients had a TPS of ≥ 50%, suggesting they may be eligible for pembrolizumab monotherapy. In the multinational KEYNOTE-001, −010, and −024 studies, which used the PD-L1 IHC 22C3 pharmDx assay, 67% and 28% of NSCLC patients exhibited PD-L1 TPS of ≥ 1% and ≥ 50%, respectively.^[17]^ Although the rates observed in these clinical trial cohorts are slightly higher compared to the current study, our results are consistent with the rates reported in the Asia Pacific subpopulation of the global EXPRESS study, which used the 22C3 pharmDx assay, where TPS ≥ 1% and ≥ 50% were reported at 53% and 25%, respectively.^[18]^ The difference in PD-L1 expression rates between our study and the clinical trial cohorts could potentially be explained by the higher prevalence of *EGFR*-mutated adenocarcinoma in Chinese NSCLC patients, as this subset is known to have lower PD-L1 levels.

The literature contains conflicting data on the association between NSCLC histology and PD-L1 expression, with some reports finding no significant differences,^[19]^ while others report higher expression in adenocarcinoma,^[8,20]^ or higher expression in squamous cell carcinoma.^[10,16]^ In the present study, PD-L1 expression was more frequently observed in squamous cell carcinomas than in adenocarcinomas, particularly in the TPS ≥ 50% subgroup. This finding may help explain the results of the CHECKMATE-078 trial, which enrolled 504 NSCLC patients (451 from China), and showed that nivolumab extended overall survival compared to docetaxel in patients with squamous (hazard ratio [HR] = 0.61) and non-squamous (HR = 0.76) NSCLC.^[21]^ Taken together, these findings suggest that squamous cell carcinoma may exhibit better sensitivity to anti-PD-1/PD-L1 agents than adenocarcinomas in Chinese NSCLC.

*EGFR* mutations are the most frequent driver mutations in Chinese NSCLC, and a series of clinical studies have suggested that ICIs may have limited efficacy in *EGFR*-mutant NSCLC.^[22,23]^ Nonetheless, the relationship between *EGFR* mutation status and PD-L1 expression remains controversial.^[16,24]^ In the current study, 28.5% of patients with PD-L1 expression carried mutated *EGFR*, whereas patients with wild-type *EGFR* showed higher positive and high-positive PD-L1 expression rates and higher TMB. Another study revealed that tumors carrying mutated *EGFR* tended to have low PD-L1 and CD8 + T cell levels, resulting in weaker immunogenicity.^[25]^ This may partially explain why NSCLC patients with mutant *EGFR* respond poorly to ICIs. In our cohort, 11.3% of the *EGFR*-mutated patients had a PD-L1 TPS of ≥ 50%. A previous study found that high PD-L1 expression in NSCLC patients with mutant *EGFR* was associated with de novo resistance to EGFR-TKIs (tyrosine kinase inhibitors).^[26]^ Our study showed that the majority (86.4%) of observed EGFR mutations were accounted for by exon 19 deletions and exon 21 L858R substitutions. In another study, it was found that compared with wild-type *EGFR*, exon 19 deletions were associated with significantly worse response rates and progression-free survival (PFS) after anti-PD-1/PD-L1 therapy in NSCLC patients, and the outcomes for patients carrying L858R *EGFR* and wild-type *EGFR* were similar,^[27]^ which may be explained by the correlation of PD-L1 expression and different *EGFR* mutation types in our study. PD-L1 high-positive expression was lower in patients with the *EGFR* T790M mutations observed in our study. A recent report found that NSCLC patients with wild-type *EGFR* who were treated with nivolumab after disease progression during *EGFR*-TKI therapy had longer PFS than patients carrying the T790M mutation (median PFS: 2.1 vs 1.3 months; *P* = .099), possibly due to lower PD-L1 expression in patients carrying the T790M mutation.^[28]^ While NSCLC patients with L858R or rare *EGFR* subtype mutations who exhibited high levels of PD-L1 may show a potential benefit from anti-PD-1/PD-L1 therapy, it is crucial to recognize that multiple factors, such as TMB, the presence of specific immune cell populations, can influence the effectiveness of immune therapy. Further larger-scale, prospective research is needed to understand the interplay between these factors and evaluate the clinical implications of these findings.

Mutations in *KRAS* or *MET* were associated with high PD-L1 expression in our cohort, with high PD-L1 levels being 3 times more common in patients carrying mutant *MET* than wild-type *MET. KRAS* or *MET* mutations were also associated with higher TMB, indicating that mutations in these genes may promote tumor immunogenicity in NSCLC. A meta-analysis noted that ICIs improved the overall survival of previously treated NSCLC patients with mutant *KRAS* (HR = 0.64; *P* = .03), but not those with wild-type *KRAS*.^[29]^ Different *KRAS* mutations encode proteins with distinct functions that influence downstream signaling. Patients carrying *KRAS* G12C and G13D mutations expressed higher levels of PD-L1 in the present study, suggesting they may show a superior response to immunotherapy. Maybe prospective trials will be necessary to determine whether *MET* and *KRAS*-TKIs as combination options with PD-L1/PD-1 inhibitors. In contrast, we found no association between PD-L1 expression level and *BRAF V600E* mutations o*r ALK* and *ROS1* translocations.

Amplification of the chromosomal region 9p24.1, which contains genes coding PD-L1 (*CD274*), PD-L2 (*PDCD1LG2*), and JAK2 (*JAK2*), has been linked to the overexpression of PD-L1 in cancer.^[30]^ The current investigation reports that 3 patients carried *CD274* and *PDCD1LG2* amplifications without other driver mutations, and all 3 expressed high PD-L1 levels. These findings imply that PD-L2 might also have predictive value for immunotherapy in NSCLC patients.

Although the association between PD-L1 expression and clinicopathological characteristics in NSCLC has been studied extensively, the question remains controversial. Our data demonstrated that high PD-L1 expression was significantly associated with female gender, biopsied tumor samples, metastatic tumor samples, and freshly prepared FFPE samples. The differential expression of PD-L1 in primary and metastatic sites is widely acknowledged. One study found that PD-L1 expression was less common in primary NSCLC tumor samples compared with metastases measured both in histologic and cytological cohorts.^[31]^ These data suggest that assessing PD-L1 expression in freshly collected tissue and from metastatic sites before treatment with anti-PD-1/PD-L1 agents is important.

There are several limitations to our study. Firstly, it is important to note that our study design is retrospective, which inherently introduces limitations in terms of data collection and potential biases in patient selection. Second, the International Association for the Study of Blueprint Project^[32]^ suggests that the Dako 22C3 and Ventana SP263 assays are equivalent; however, using 2 assays in the present study may have affected the expression estimates. Finally, some data on baseline characteristics were missing or incomplete.

## 5. Conclusions

In conclusion, PD-L1 expression in Chinese NSCLC varied in terms of sampling methods, histologic types, oncogene, and PD-L1 (*CD274*)/PD-L2 (*PDCD1LG2*) alteration status. Given the association with PD-L1 expression, our findings support the potential for combination immunotherapy with *MET, KRAS* inhibitors, and *CD274/PDCD1LG2* amplification, which may support eligibility for treatment with anti-PD-1/PD-L1 agents for patients with NSCLC.

## Acknowledgments

We thank 3D Medicines Inc. for its cooperation with this study. They provided the NGS panel detection and the annotations of the results.

## Author contributions

**Conceptualization:** Jindong Guo, Ying Zhou, Liwen Xiong.

**Data curation:** Jindong Guo, Haibin Yuan, Bei Zhang, Ying Zhou, Liwen Xiong.

**Formal analysis:** Haibin Yuan, Bei Zhang, Ding Zhang, Liwen Xiong.

**Methodology:** Bei Zhang.

**Project administration:** Haibin Yuan, Bei Zhang, Ding Zhang.

**Resources:** Haibin Yuan, Bei Zhang.

**Validation:** Jindong Guo, Haibin Yuan, Ding Zhang, Liwen Xiong.

**Visualization:** Bei Zhang, Ding Zhang.

**Writing – original draft:** Yimin Zhu, Zhiyuan Che, Ding Zhang.

**Writing – review & editing:** Yimin Zhu, Zhiyuan Che.

## Supplementary Material








