# Supplementary figures and images for: PD-L1 expression and its correlation with clinicopathological and molecular characteristics in Chinese patients with non-small cell lung cancer
Source: Medicine (Baltimore). 2024 Feb 23;103(8):e36770. doi: 10.1097/MD.0000000000036770 (PMC11309668; doi:10.1097/MD.0000000000036770)

## Slide 1
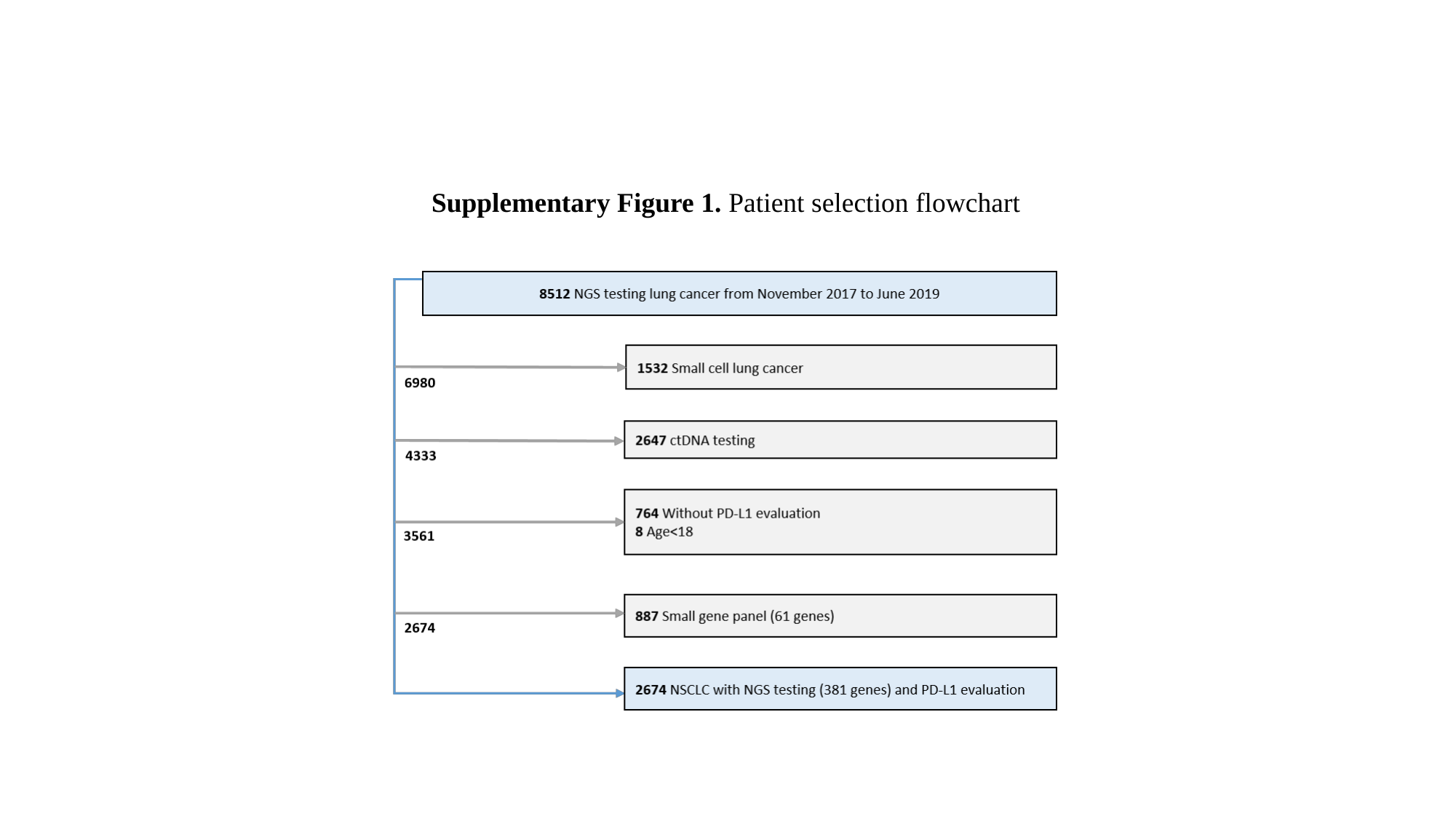

Supplementary Figure 1. Patient selection flowchart

Supplement: Supplementary file 1 [file medi-103-e36770-s001.pptx]

## Slide 1
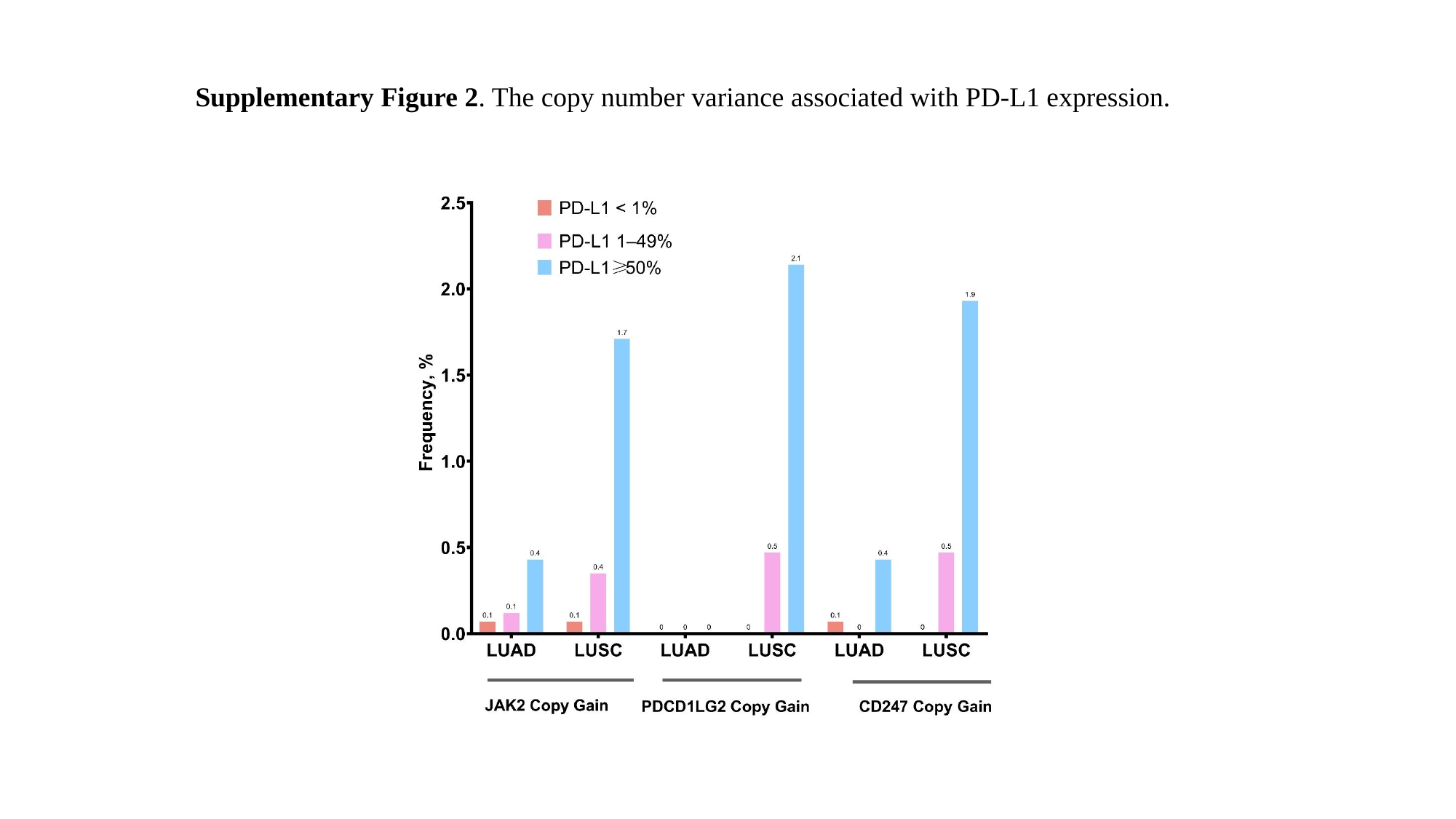

Supplementary Figure 2. The copy number variance associated with PD-L1 expression.

Supplement: Supplementary file 2 [file medi-103-e36770-s002.pptx]

## Slide 1
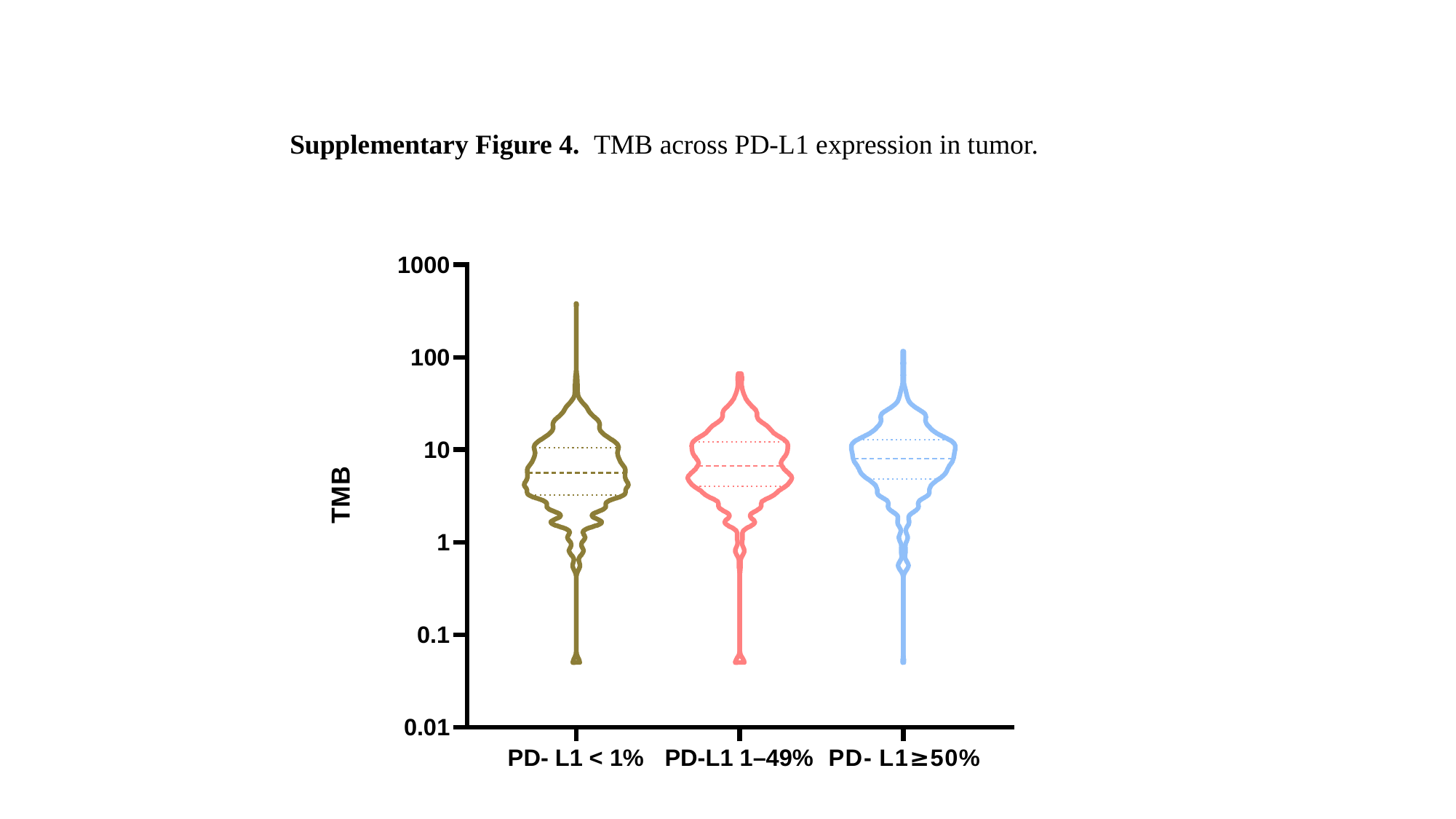

Supplementary Figure 4. TMB across PD-L1 expression in tumor.

Supplement: Supplementary file 4 [file medi-103-e36770-s004.pptx]
