# Supplementary material for: PD-L1 expression and its correlation with clinicopathological and molecular characteristics in Chinese patients with non-small cell lung cancer
Source: Medicine (Baltimore). 2024 Feb 23;103(8):e36770. doi: 10.1097/MD.0000000000036770 (PMC11309668; doi:10.1097/MD.0000000000036770)
Supplement: Supplementary file 3 [file medi-103-e36770-s003.pptx]

## Slide 1
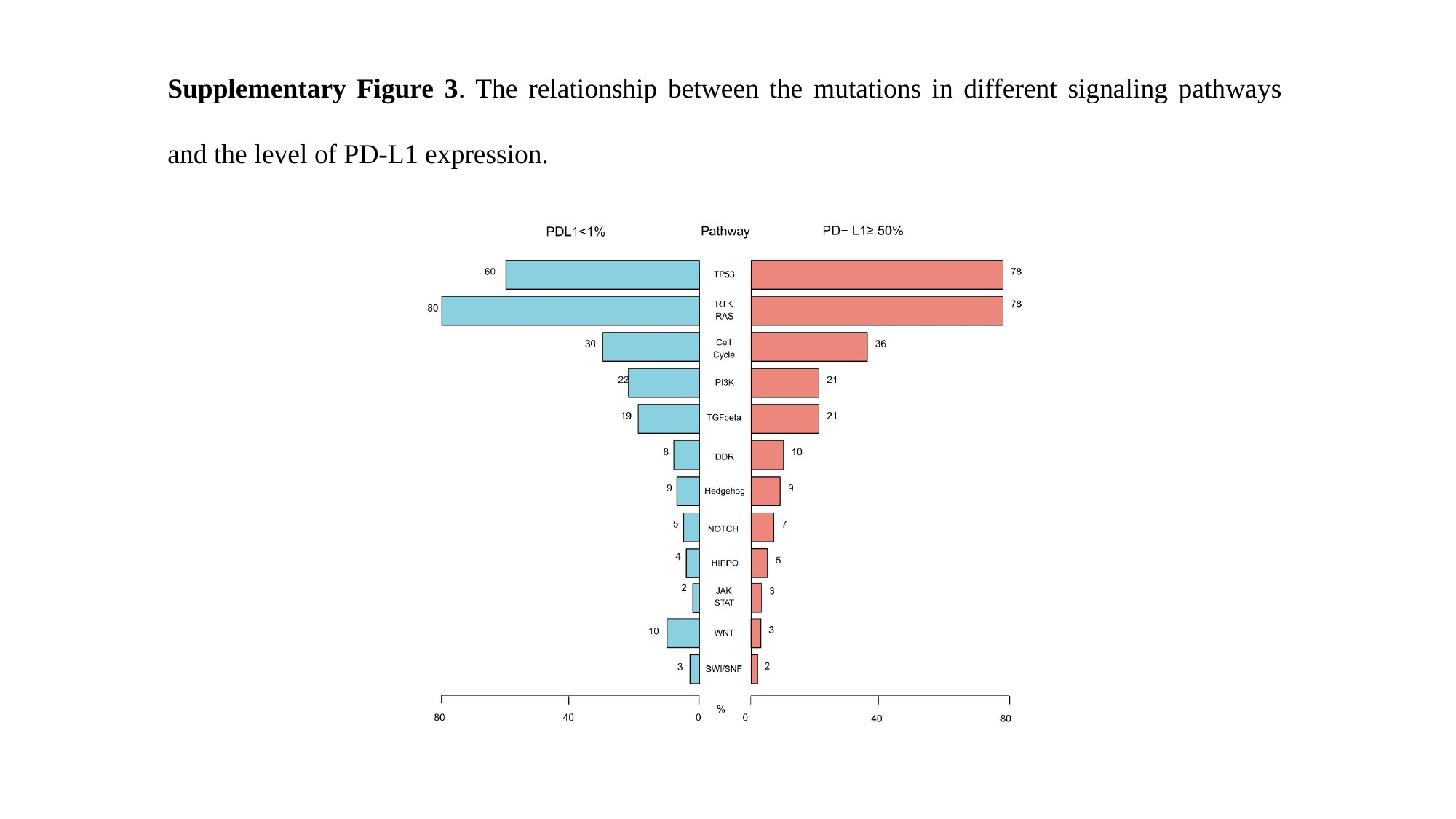

Supplementary Figure 3. The relationship between the mutations in different signaling pathways and the level of PD-L1 expression.
